# Co-Efficient Vector Based Differential Distributed Quasi-Orthogonal Space Time Frequency Coding

**DOI:** 10.3390/s23177540

**Published:** 2023-08-30

**Authors:** Nnamdi Nwanekezie, Oluyomi Simpson, Gbenga Owojaiye, Yichuang Sun

**Affiliations:** School of Physics, Engineering and Computer Science (SPECS), University of Hertfordshire, College Lane Campus, Hatfield AL10 9AB, UK; n.c.nwanekezie@herts.ac.uk (N.N.); y.sun@herts.ac.uk (Y.S.)

**Keywords:** differential distributed space time frequency coding, co-efficient vectors, unitary matrices, quasi-orthogonal codes

## Abstract

Distributed space time frequency coding (DSTFC) schemes address problems of performance degradation encountered by cooperative broadband networks operating in highly mobile environments. Channel state information (CSI) acquisition is, however, impractical in such highly mobile environments. Therefore, to address this problem, designers focus on incorporating differential designs with DSTFC for signal recovery in environments where neither the relay nodes nor destination have CSI. Traditionally, unitary matrix-based differential designs have been used to generate the differentially encoded symbols and codeword matrices. Unitary based designs are suitable for cooperative networks that utilize the amplify-and-forward protocol where the relay nodes are typically required to forego differential decoding. In considering other scenarios where relay nodes are compelled to differentially decode and re-transmit information signals, we propose a novel co-efficient vector differential distributed quasi-orthogonal space time frequency coding (DQSTFC) scheme for decode-and-forward cooperative networks. Our proposed space time frequency coding scheme relaxes the need for constant channel gain in the temporal and frequency dimensions over long symbol periods; thus, performance degradation is reduced in frequency-selective and time-selective fading environments. Simulation results illustrate the performance of our proposed co-efficient vector differential DQSTFC scheme under different channel conditions. Through pair-wise error probability analysis, we derive the full diversity design criteria for our code.

## 1. Introduction

The fundamental idea behind space–time–frequency (STF) coding in cooperative networks is to provide a scheme through which spatial, temporal, and frequency coding is exploited. This has led to the design of distributed space–time–frequency coding (DSTFC) schemes. In DSTFC schemes, elements of the codeword matrices are simultaneously transmitted via relay nodes across multiple orthogonal frequency-division multiplexing (OFDM) time slots and frequency sub-carriers. The principal objective is to design a coding scheme that mitigates the performance degradation experienced by distributed space time coding–orthogonal frequency division multiplexing (DSTC-OFDM) schemes and distributed space–frequency coding (DSFC) schemes in severe time-selective and frequency-selective fading channels, respectively. Furthermore, in the DSTC-OFDM schemes of [1,2,3] and the DSFC schemes of [4,5,6,7,8], for example, the channel gain on adjacent OFDM [9,10] time slots and frequency sub-carriers, respectively, is assumed to remain quasi-static to facilitate signal recovery at the destination. This assumption is impractical when signals with long symbol duration are transmitted or in cases involving large numbers of cooperating nodes. However, in the scheme proposed by this study, it is shown that DSTFC can be designed to mitigate this problem by simultaneously transmitting across OFDM time-slots and frequency sub-carriers. Therefore, this ensures that the requirement for constant channel gain in the temporal and frequency dimensions is more relaxed.

In order to implement cooperation in multi-hop networks generally, the most popular relaying protocols currently being used are the Decode-and-Forward (DF) [11,12] and Amplify-and-Forward (AF) [13,14] protocols. In the bid to cater for highly mobile environments where the nodes are unable to acquire channel state information (CSI), research efforts have been focused on the implementation of differential schemes for data transmission in non-coherent cooperative networks [15]. For example, a randomized differential scheme was proposed for a partially coherent cooperative network in [11] where the destination has partial CSI as opposed to full CSI. In other words, the source node is assumed to have CSI for the source–relay link while that of the relay–destination link cannot be acquired. The research in [16] provides the differential strategy for non-constant modulus constellations whereby the differential procedure is dependent on both amplitude and phase transitions. Furthermore, in their work, they focused on the reduction of the computational complexity of the differential procedure. Similarly, the studies carried out in [15,17,18] consider differential orthogonal coding schemes where neither the relays nor the destination are able to acquire CSI.

Fundamentally, when differential strategies are incorporated with coding schemes, a principal aspect is how to generate the differentially encoded symbols and, subsequently, the transmission matrix (at the source and relay nodes, respectively) in such a manner that would ensure their recovery at the destination without the need for CSI. Differential strategies can be implemented in cooperative networks through the utilisation of differential unitary matrix design as adopted by the authors in all the aforementioned works [17,19,20,21,22]. In contrast to existing works in the literature [23], differential strategies can also be implemented in cooperative networks through the use of a differential co-efficient vector design, which is being proposed in this study. It is evident from [17,24,25] that differential distributed space time frequency coding (DSTBC) schemes adopting the unitary matrix design are amenable to the AF protocol. This is because the relay nodes are generally required to forego CSI acquisition such that differential decoding is only performed at the destination. However, in contrast, for practical scenarios where the relay nodes are compelled to differentially decode and re-transmit information signals received from the source node, the co-efficient vector design is better suited. The co-efficient vector design reflects its utilisation specifically for applications like Adhoc and sensor networks that require inherent data correlation at intermediate nodes. The integration of differential schemes with DSTFC in broadband networks is even more challenging because of the presence of multiple paths in frequency-selective fading channels and multiple broadcast phases between the source and destination.

Furthermore, all the aforementioned works employ orthogonal codes and are thus only capable of providing full code-rate and full diversity for a maximum of two-relay cooperative networks. In order to cater for high data rate in cooperative networks, our proposed scheme employs rotated constellation quasi-orthogonal codes, which achieves full code-rate for any number of relay nodes. In addition, we show how the quasi-orthogonal codes are constructed from a proper choice of constellation sets. Moreover, the full mapping scheme and differential recipe for utilizing co-efficient vectors in cooperative networks are presented.

Motivated by all the aforementioned, we propose co-efficient vector differential distributed quasi-orthogonal space–time–frequency coding (DQSTFC) for cooperative networks. This study thus aims to make the following contributions:

- Due to the involvement of multiple broadcast phases in cooperative networks, our proposal of designing the STF codes at the source node aims to simplify the operation of relay nodes. Here, the codewords are carefully distributed in the temporal and frequency dimensions to relax the assumption that the channel is quasi-static in the time and frequency domains for long symbol periods;
- We provide a systematic construction of full rate quasi-orthogonal codes that can exploit full STF diversity and non-coherent signal detection. The full differential procedure using our proposed co-efficient vector design is also provided in this work. Through pair-wise error probability analysis, we derive the necessary conditions for our code to achieve full STF diversity while the coding gain is maximized as much as possible;
- The simulation carried out also provides an analytical study of the performance of our co-efficient vector differential DQSTFC scheme in frequency-selective fading and time-selective fading environments. We also compare and distinguish between the unitary matrices and co-efficient vector designs in terms of Bit Error Rate (BER) performance thus highlighting the robustness of our proposed non-coherent scheme against highly selective fading environments. In addition, we generalize the co-efficient vector design to cooperative networks with four, six, and eight relay nodes.

The remaining part of this paper is organised as follows: Section 2 presents the cooperative system and channel models and showing how the STF codes are designed at the source node and the relay nodes. Furthermore, Section 3 covers the differential encoding and decoding procedure using our proposed co-efficient vector design while Section 4 presents the pair-wise error probability analysis. Finally, Section 5 presents some simulation results and Section 6 contains the conclusion.

*Notation*: A bold-face upper case letter denotes a matrix while a bold-face lower case letter denotes a vector; ${\left(\cdot \right)}^{*}$,${\left(\cdot \right)}^{T}$,${\left(\cdot \right)}^{H}$ denote conjugate, transpose, and conjugate-transpose, respectively; A⨀B denotes the Hadamard product or entry-wise product of the matrices A and B; A⨂B denotes the Kronecker product of the matrices A and B; $tr\left(\cdot \right)$ is a trace function; $E\left(\cdot \right)$ and $var\left(\cdot \right)$ represent expectation and variance of a random variable, respectively; ${\left\|X\right\|}_{F}$ denotes the Frobenius norm of the matrix X; $\left|x\right|$ denotes the absolute value of x; $det\left(X\right)$ stands for the determinant of X; $diag\left(\left[x_{0},x_{1},\ldots ,x_{N-1}\right]\right)$ denotes an N×N diagonal matrix with diagonal entries $x_{0},x_{1},\ldots ,x_{N-1};\left\lfloor x\right\rfloor$ denotes the largest integer smaller than x; $I_{N}$ is an N×N identity matrix; and finally, superscript $C^{T\times N}$ gives the dimension of a complex matrix.

## 2. Distributed Space–Time–Frequency Coding

### 2.1. System Model

**Figure 1:**
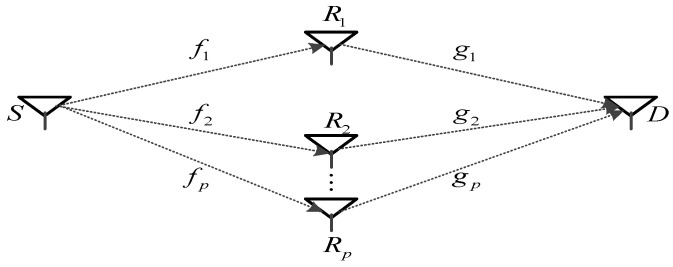
P-relay Cooperative Network.

The cooperative network consists of a source node, a destination node, and P relay nodes as shown in Figure 1. Each node is equipped with a single antenna, which is used for both transmission and reception. The transmission from the source node to the destination is divided into the ‘transmit’ and ‘cooperate’ stages. In the ‘transmit’ stage, the source node sends information signals to the cooperating relay nodes while in the ‘cooperate’ stage, the source node keeps silent and the cooperating relay nodes decode and forward the information signals to the destination. We address the problem of differential encoding and decoding where the relay nodes and the destination are unable to acquire CSI. Our investigation in this work is carried out under the assumption of perfect inter-relay synchronization. It is noteworthy that the use of cyclic prefix can provide robustness against synchronization errors at the relays. This benefit, which is owed to the employment of OFDM transmission, is applicable to our proposed DQSFC scheme. This assumption is, however, critical in practice due to the distributive nature of relays in space. Asynchronous transmission of the relays may result in degradation in diversity gain. Specifically, the impact of synchronization errors have been studied in [26] based on analytical and simulation results. Unlike STF coding in multiple-antenna systems, STF coding in cooperative networks must be implemented in two distinct stages, namely coding at the source node and coding at the relay nodes. We first describe how the coded data is designed at the source node.

### 2.2. Space Time Frequency Coding at the Source Node

The cooperative system is based on OFDM modulation with N sub-carriers and T OFDM time slots. At the source node, a stream of NT modulated symbols, $s=\left[s\left(0\right),s\left(1\right),\ldots ,s\left(NT-1\right)\right]$, are generated from an MPSK constellation. The NT symbols are simultaneously distributed across n=0,1,…,N−1 sub-carriers and t=0,1,…,T−1 OFDM time slots. In the frequency dimension, the N sub-carriers are grouped into $K=\left\lfloor N/N_{b}\right\rfloor$ blocks. Similarly, in the temporal dimension, the T OFDM time slots are grouped into $K=\left\lfloor T/T_{b}\right\rfloor$ blocks. Thus, each $k_{th}$ block, k=1,2,…,K is of length $\Gamma =N_{b}T_{b}$, where $N_{b}$ and $T_{b}$ denote the number of frequency sub-carriers and time slots, respectively, per block. Based on this, the coded source node data is constructed:

(1) $$x=\left[x\left(0\right),x\left(1\right),\ldots ,x\left(NT-1\right)\right]={\left[x_{1}^{T},x_{2}^{T},\ldots ,x_{K}^{T},0_{NT-KP\Gamma }^{T}\right]}^{T}$$

where $x_{k}={\left[x_{k}(1),\ldots ,x_{k}(P\Gamma )\right]}^{T}\in C^{P\Gamma \times 1}$ and $\left(NT-KP\Gamma \right)$ zeros are padded into x if NT is not an integer multiple of KPΓ. The coded source node data can be viewed as a concatenation of information symbols in the temporal and frequency dimensions since the elements of $x_{k}$ are stacked simultaneously unto ${PN}_{b}$ adjacent data sub-carriers and ${PT}_{b}$ OFDM time slots. Assuming the system is perfectly synchronized in the time and frequency domains, the PΓ×1 data vector transmitted by the source node in the *k*th block can be written as $x_{k}={\left[x_{k,t,n}\left(1\right),\ldots ,x_{k,t,n}\left(P\Gamma \right)\right]}^{T}\in C^{P\Gamma \times 1}$, where $x_{k,t,n}\left(i\right),i=1,2,\ldots ,P\Gamma$ denotes the *i*th symbol transmitted on the $n_{th}$ sub-carrier within the $t_{th}$ OFDM time slot. Since the source node transmits the data vector $x_{k}$ to P relay nodes simultaneously across ${PT}_{b}$ OFDM time slots and ${PN}_{b}$ frequency sub-carriers, then the source node code is capable of achieving a diversity of order $\Gamma =N_{b}T_{b}$ at each relay node. The criteria for achieving this diversity order will be clarified further on in this paper. The elements of x are normalized such that $E\left({\left|x_{k}\right|}^{2}\right)=1$.

### 2.3. Multipath Channel Model

The channel at the *t*th OFDM time slot between the source node and the *p*th relay node is described by the impulse response vector $f_{p,t}={\left[f_{p,t}(0),\ldots ,f_{p,t}(L_{SR}-1)\right]}^{T}$. Similarly, the channel at the *t*th OFDM time slot between the *p*th relay node and the destination is described by the impulse response vector $g_{p,t}={\left[g_{p,t}(0),\ldots ,g_{p,t}(L_{RD}-1)\right]}^{T}$, where $L_{SR}$ and $L_{RD}$ denote the number of independent channel taps on the source–relay link and relay–destination link, respectively. The multipath fading channel between the source node and the $p_{th}$ relay node is modelled as $f_{p}\left(t\right)=\sum_{l=0}^{L_{SR}-1}f_{p,t}\left(l\right)\delta \left(t-\alpha_{l}\right)$. Similarly, the multipath fading channel between the $p_{th}$ relay node and the destination is modelled as $g_{p}\left(t\right)=\sum_{l=0}^{L_{RD}-1}g_{p,t}\left(l\right)\delta \left(t-\beta_{l}\right)$, where the complex amplitudes $f_{p,t}\left(l\right)$ and $g_{p,t}\left(l\right)$ are assumed to be independent zero-mean complex Gaussian random variables with variances $E\left({\left|f_{p,t}\left(l\right)\right|}^{2}\right){=\sigma }_{SR}^{2}(l)$ and $E\left({\left|g_{p,t}\left(l\right)\right|}^{2}\right)=\sigma_{RD}^{2}\left(l\right),$ respectively. The delay of the $l_{th}$ path is denoted by $\alpha_{l}$ and $\beta_{l}$ while $\delta \left(\cdot \right)$ is the Dirac delta function.

### 2.4. Space Time Frequency Coding at the Relay Nodes

If the data vector generated by the source node is of the form $\sqrt{E_{S}P\Gamma }x_{k}$, where $E_{S}$ denotes average transmit-power, the signal received at the $p_{th}$ relay node in the $k_{th}$ block after cyclic prefix (CP) removal and fast Fourier transform (FFT) demodulation is given in vector form by:

(2) $$r_{k,p}=\sqrt{E_{S}P\Gamma }x_{k}⊙f_{k,p}+n_{k,p}$$

where r_(k,p)=[r_(k,p)(1),…,r_(k,p)(Γ),…,r_(k,P)(1),…,r_(k,P)(Γ)]^T∈C^(PΓ×1),f_(k,p)=[f_(k,p,t,n)(1),…,f_(k,p,t,n)(PΓ)]^T, $f_{k,p,t,n}\left(i\right)$ is the channel gain at the *n*th sub-carrier of the *p*th relay node during the *t*th time slot, and $n_{k,p}={\left[n_{k,p}\left(1\right),\ldots ,n_{k,p}\left(\Gamma \right),\ldots ,n_{k,P}\left(1\right),\ldots ,n_{k,P}\left(\Gamma \right)\right]}^{T}$ is the zero-mean complex Gaussian noise vector with covariance $N_{0}I_{P\Gamma }$. The multipath fading channel $f_{p,k,t,n}\left(i\right)$ between the source node and the $p_{th}$ relay node is modelled as $f_{p,k,t,n}\left(i\right)=\sum_{l=0}^{L_{SR}-1}f_{p,t}(l)e^{-j2\pi ln/N}={f_{p,t}}^{T}\omega$, $\omega ={\left[1,e^{-j2\pi n/N},\ldots ,e^{-j2\pi Ln/N}\right]}^{T}$. The average signal-to-noise ratio (SNR) of the channel between the source node and the *p*th relay node is given by $\Upsilon_{SR}=E_{S}P\Gamma /N_{0}$.

The procedures through which the STF codes are constructed at the relay nodes are described below. Given that the *p*th relay node receives $r_{k,p}\in C^{P\Gamma \times 1}$ in (2), it differentially decodes the received signal and obtains ${\tilde{x}}_{k}={\left[{\tilde{x}}_{k,t,n}\left(1\right),\ldots ,{\tilde{x}}_{k,t,n}\left(P\Gamma \right)\right]}^{T}$, where ${\tilde{x}}_{k}$ is the differentially decoded version of the source node signal $x_{k}$. The differential procedure is explained in the next section. In our STF coding scheme, we configure the *p*th relay node to forward a subset of the decoded signal ${\tilde{x}}_{k}$, which we define as ${\tilde{\bar{x}}}_{k}={\left[{\tilde{\bar{x}}}_{k,p,t,n}\left(1\right),\ldots ,{\tilde{\bar{x}}}_{k,p,t,n}\left(\Gamma \right)\right]}^{T}\in C^{\Gamma \times 1}$, while the remaining $\left(P\Gamma -\Gamma \right)$ symbols are discarded. Note that ${\tilde{\bar{x}}}_{k,p,t,n}\left(i\right)$ denotes the *i*th symbol transmitted by the *p*th relay node on the *n*th sub-carrier of the *t*th OFDM time slot in the *k*th block. Based on this, the received signal at the *p*th relay node can be rewritten as follows:

(3) $${\bar{r}}_{k,p}=\sqrt{E_{S}P\Gamma }{\bar{x}}_{k}⊙{\bar{f}}_{k,p}+{\bar{n}}_{k,p}$$

where ${\bar{x}}_{k}={\left[x_{k}\left(1\right),\ldots ,x_{k}\left(\Gamma \right)\right]}^{T}$, ${\bar{f}}_{k,p}={\left[f_{k,p,t,n}\left(1\right),\ldots ,f_{k,p,t,n}\left(\Gamma \right)\right]}^{T}$ and ${\bar{n}}_{k,p}={\left[n_{k,p}\left(1\right),\ldots ,n_{k,p}\left(\Gamma \right)\right]}^{T}$. In our DQSTFC scheme, the P relay nodes are designed to construct Γ×P quasi-orthogonal signal matrices at the destination. In order to achieve this, each *p*th relay node is equipped with a Γ×Γ unitary matrix $M_{p}$, which we refer to as the ‘relay matrix’. The relay matrix is a matrix of 1 s and 0 s, which enable the relay nodes to generate codewords with a quasi-orthogonal structure at the destination. The structure of the relay matrix is given in Sections 3 and 4 of [27] for cooperative networks with different number of relay nodes. Specifically, it is assumed that J relay nodes are programmed to multiply their relay matrix by the decoded signal ${\left[{\tilde{\bar{x}}}_{k,p,t,n}\left(1\right),\ldots ,{\tilde{\bar{x}}}_{k,p,t,n}\left(\Gamma \right)\right]}^{T}$ while the remaining P−J relay nodes are programmed to multiply their relay matrix by the conjugate of the received signal ${\left[{{\tilde{\bar{x}}}_{k,p,t,n}\left(1\right)}^{*},\ldots ,{{\tilde{\bar{x}}}_{k,p,t,n}\left(\Gamma \right)}^{*}\right]}^{T}$. Thus, in the *k*th block, the *p*th relay node transmits a Γ×1 vector $t_{k,p}$ given by:

(4) $$t_{k,p}=\sqrt{\frac{E_{C}}{E_{S}+1}}M_{p}{\tilde{\bar{x}}}_{k},{\tilde{\bar{x}}}_{k}\in \left\{{\left[{\tilde{\bar{x}}}_{k,p,t,n}\left(1\right),\ldots ,{\tilde{\bar{x}}}_{k,p,t,n}\left(\Gamma \right)\right]}^{T},{\left[{{\tilde{\bar{x}}}_{k,p,t,n}\left(1\right)}^{*},\ldots ,{{\tilde{\bar{x}}}_{k,p,t,n}\left(\Gamma \right)}^{*}\right]}^{T}\right\}$$

Since each *p*th relay node transmits the data vector $t_{k,p}$ to the destination on $\Gamma =N_{b}T_{b}$ sub-carriers/time slots, then all the P relay nodes can jointly achieve a diversity of order PΓ at the destination.

Assuming the relay nodes are synchronized at symbol level such that the nodes can transmit simultaneously, the signal received at the destination in the $k_{th}$ block after CP removal and FFT demodulation is given by:

(5) $$y_{k,i}=\sum_{p=1}^{P}t_{k,p}⊙g_{k,p}+z_{k,i},i=1,2,\ldots ,\Gamma$$

where $y_{k,i}={\left[y_{k,i}\left(1\right),\ldots ,y_{k,i}\left(\Gamma \right)\right]}^{T}$, $g_{k,p}={\left[g_{k,p,t,n}\left(1\right),\ldots ,g_{k,p,t,n}\left(\Gamma \right)\right]}^{T}$, and $z_{k,i}={\left[z_{k,i}\left(1\right),\ldots ,z_{k,i}\left(\Gamma \right)\right]}^{T}$ is the zero-mean complex Gaussian noise term with covariance $N_{0}I_{\Gamma }$. The frequency response of the channel at the *n*th sub-carrier between the *p*th relay node and the destination during the *t*th time slot is denoted by $g_{k,p,t,n}\left(i\right)=\sum_{l=0}^{L_{RD}-1}g_{p,t}\left(l\right)e^{-j2\pi ln/N}={g_{p,t}}^{T}\omega$. The average SNR of the channel between the $p_{th}$ relay node and the destination is given by $\Upsilon_{RD}=E_{C}/N_{0}$. Substituting for $t_{k,p}$ in (4), (5) becomes:

(6) $$y_{k,i}=\sum_{p=1}^{P}\sqrt{\frac{E_{C}E_{S}N_{C}}{E_{S}+1}}M_{p}{\tilde{\bar{x}}}_{k}⊙g_{k,p}+z_{k,i},i=1,2,\ldots ,\Gamma$$

The signal received at the destination in the *k*th block can be written in compact form as:

(7) $$Y_{k}=\sqrt{\rho }X_{k}G_{k}+Z_{k}$$

where $\rho =\frac{E_{C}E_{S}N_{C}}{E_{S}+1}$, $Y_{k}=\left[y_{k,1},\ldots ,y_{k,\Gamma }\right]\in C^{\Gamma \times \Gamma }$, $y_{k,i}={\left[y_{k,i}\left(1\right),\ldots ,y_{k,i}\left(\Gamma \right)\right]}^{T}$, $X_{k}=\left[M_{1}{\tilde{\bar{x}}}_{k},\ldots ,M_{J}{\tilde{\bar{x}}}_{k},M_{J+1}{{\tilde{\bar{x}}}_{k}}^{*},\ldots ,M_{P}{{\tilde{\bar{x}}}_{k}}^{*}\right]\in C^{\Gamma \times P}$, $G_{k}=\left[g_{k,1},\ldots ,g_{k,\Gamma }\right]\in C^{P\times \Gamma }$, $g_{k,i}={\left[g_{k,t,n}\left(1\right),\ldots ,g_{k,t,n}\left(P\right)\right]}^{T}=\left(I_{P}⊗\omega^{T}\right)g,g={\left[g_{1,t}\left(0\right),\ldots ,g_{1,t}\left(L-1\right),..,g_{P,t}\left(0\right),\ldots ,g_{P,t}\left(L-1\right)\right]}^{T}$, and $Z_{k}=\left[{\tilde{z}}_{k,1},\ldots ,{\tilde{z}}_{k,\Gamma }\right]\in C^{\Gamma \times \Gamma }$,$z_{k,i}={\left[z_{k,i}\left(1\right),\ldots ,z_{k,i}\left(\Gamma \right)\right]}^{T}$.

The P×Γ quasi-orthogonal channel matrix $G_{k}$ captures the channel coefficients between the P relay nodes and the destination. Here, it is assumed that the channel coefficient between the P relay nodes and the destination remain quasi-static, at least, across $T_{b}$ adjacent OFDM time slots and $N_{b}$ adjacent frequency sub-carriers. Therefore, this means that the channel is constant during the transmission of Γ symbols, that is, $g_{k,n}$ is constant for i=1,2,…,Γ. Thus, the requirement for constant channel gain during the transmission of Γ symbols is relaxed across Γ/2 time slots and Γ/2 sub-carriers. Further on, this paper will show the robustness of our scheme under certain fading conditions in comparison to STBC-OFDM and SFBC schemes where the channel coefficients are required to remain constant across Γ time slots and Γ sub-carriers, respectively, during the transmission of Γ symbols.

The matrix $X_{k}$ that is generated at the destination by the P relay nodes is a Γ×P quasi-orthogonal signal matrix containing either complex information symbols $\left\{{\tilde{\bar{x}}}_{k,p,t,n}\left(1\right),\ldots ,{\tilde{\bar{x}}}_{k,p,t,n}\left(\Gamma \right)\right\}$ or their conjugates $\left\{{{\tilde{\bar{x}}}_{k,p,t,n}\left(1\right)}^{*},\ldots ,{{\tilde{\bar{x}}}_{k,p,t,n}\left(\Gamma \right)}^{*}\right\}$. Thus, $X_{k}$ in (7) can be rewritten as $X_{k}=\left[{{\tilde{\bar{x}}}_{k,1}}^{T},\ldots ,{{\tilde{\bar{x}}}_{k,P}}^{T}\right]\in C^{\Gamma \times P},{\tilde{\bar{x}}}_{k,p}=\left[{\tilde{x}}_{k,t,n}\left(1\right),\ldots ,{\tilde{x}}_{k,t,n}\left(\Gamma \right)\right]$, where ${\tilde{\bar{x}}}_{k,p}$ is the *p*th column of $X_{k}$. In other words, the *p*th relay node transmits the *p*th column vector of $X_{k}$. In order to recover information symbols at the destination without CSI, two consecutive quasi-orthogonal signal matrices $X_{k}$ and $X_{k+1}$ must be received at the destination in the *k*th block and (*k* + 1)th block, respectively. The first signal matrix $X_{k}$ is termed the ‘reference’ quasi-orthogonal matrix because it is only required for differential decoding and, thus, contains no valid data while the subsequent quasi-orthogonal signal matrix $X_{k+1}$ conveys the valid data. From the structure of $X_{k}$, it is clear that the codeword guarantees one complex-valued symbol per time slot/per sub-carrier use when the relay nodes transmit to the destination, which is referred to here as a full code rate.

## 3. Co-Efficient Vector Differential Procedure

### 3.1. Co-Efficient Vector Generator

**Figure 2:**
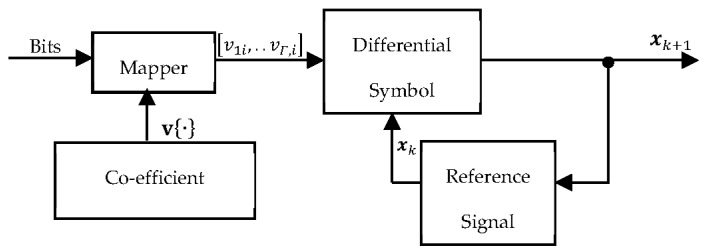
Co-efficient Vector Differential Encoder.

Our proposed co-efficient vector differential procedure is explained in this section. The design typically comprises three sub-systems, including the co-efficient vector generator, the mapper, and the feedback sub-system as illustrated in Figure 2. Given Γm input bits, the source node constructed a length $2^{\Gamma m}$ co-efficient vector set given by $v=\left\{{\left[v_{1,1},{\ldots ,v}_{\Gamma ,1}\right]}^{T},{\left[v_{1,2},{\ldots ,v}_{\Gamma ,2}\right]}^{T},\ldots ,{\left[v_{1,2^{2m}},\ldots ,v_{\Gamma ,2^{2m}}\right]}^{T}\right\}.$ The co-efficient vector set v was made up of $2^{2m}$ unit-length distinct vectors ${\left[v_{1,d},{\ldots ,v}_{\Gamma ,d}\right]}^{T},d\in \left\{1,2,\ldots ,2^{2m}\right\}$; m was the spectral efficiency. Then, a pseudo-random one-to-one mapping scheme $P\left(.\right)$ was defined for any $m={log}_{2}M$ M-PSK signal constellation such that the Γm input bits were mapped onto v. Note that M was the constellation size. The Γm input bits arriving at the encoder in the ${\left(k+1\right)}_{th}$ block were used to select the corresponding Γ×1 vector from the co-efficient vector set v. In other words, the co-efficient vector selected from v was exclusively dependent on the input bits generated and the pseudo-random one-to-one mapping scheme $P\left(.\right)$. Given any random set of input bits, the selected co-efficient vector could be written as ${{\bar{v}}_{k+1}=\left[v_{1},{\ldots ,v}_{\Gamma }\right]}^{T}$. The elements of ${\bar{v}}_{k+1}$, given by $\left\{v_{1},{\ldots ,v}_{\Gamma }\right\}$, represented the valid transmitted information symbols that had to be recovered at the destination without CSI.

This section may be divided by subheadings. It should provide a concise and precise description of the experimental results, their interpretation, and the experimental conclusions that can be drawn.

### 3.2. Quasi-Orthogonal Code Construction

We now show how the elements of the co-efficient vector ${\bar{v}}_{k+1}$ were rotated to ensure quasi-orthogonality. Given that the stream of Γm input bits were mapped into Γ symbols denoted by $v_{i},i=1,2,\ldots ,\Gamma$, the symbols were then combined as $v_{1}=v_{1}+{\tilde{v}}_{3}$, $v_{2}=v_{2}+{\tilde{v}}_{4}$, $v_{3}=v_{1}-{\tilde{v}}_{3},v_{4}=v_{2}-{\tilde{v}}_{4}$, and so on. Let $\Phi =D\cdot diag\left[1,e^{{j\theta }_{1}},\ldots ,e^{{j\theta }_{\left(\Gamma /2\right)-1}}\right]$, where D was a $\left(\Gamma /2\right)\times \left(\Gamma /2\right)$ Hadamard matrix, the information symbols were then constructed as [v_(k+1)(1),v_(k+1)(3),…,v_(k+1)(Γ−1)]^T=Φ⋅[v_1,v_3,…,v_(Γ−1)]^T and [v_(k+1)(2),v_(k+1)(4),…,v_(k+1)(Γ)]^T=Φ⋅[v_2,v_4,…,v_Γ]^T. Thus, ${\left[v_{k+1}\left(1\right),v_{k+1}\left(2\right),\ldots \right]}^{T}$ were mapped onto a signal constellation A of size $2^{m}$ while ${\left[v_{k+1}\left(3\right),v_{k+1}\left(4\right),\ldots \right]}^{T}$ were mapped onto a signal constellation $A_{r}$, which was a rotated version of A. The rotation angles θ of the information symbols ensured that the codes achieve full diversity, see Chapter 5 of [28] for further explanation on constellation rotation.

### 3.3. Differential Encoding Using Co-Efficient Vectors

The STF coded data vector $x_{k+1}$ to be transmitted by the source node in the (*k* + 1)th block was generated from the STF coded data vector $x_{k}={\left[x_{k}(1),\ldots ,x_{k}(P\Gamma )\right]}^{T}$ transmitted by the source node in the *k*th block. We first discussed how the data vector in the (*k* + 1)th block was generated using the co-efficient vector and the information signals transmitted in the *k*th block. The first step was for the source node to compute the Γ×1 data vector ${\bar{x}}_{k+1}$ for the (*k* + 1)th block using the transmitted information signals in the *k*th block and the selected co-efficient vector as follows:

(8) $${\bar{x}}_{k+1}=X_{k}{\bar{v}}_{k+1}$$

where ${\bar{v}}_{k+1}={\left[v_{1,i},{\ldots ,v}_{\Gamma ,i}\right]}^{T}$ and $X_{k}$ was the ‘reference’ quasi-orthogonal signal matrix that was generated by the P relay nodes in the *k*th block. It was assumed that the source node had prior knowledge of the relay matrices $\left\{M_{1},\ldots {,M}_{J},M_{J+1},\ldots ,M_{P}\right\}$. The source node also knew ${\bar{x}}_{k}={\left[x_{k}\left(1\right),\ldots ,x_{k}\left(\Gamma \right)\right]}^{T}$; hence, it could compute $X_{k}=\left[M_{1}{\bar{x}}_{k},\ldots ,M_{J}{\bar{x}}_{k},M_{J+1}{{\bar{x}}_{k}}^{*},\ldots ,M_{P}{{\bar{x}}_{k}}^{*}\right]$ in (8). Then, finally, using ${\bar{x}}_{k+1}$ in (8), the source node constructed the PΓ×1 data vector $x_{k+1}={\left[x_{k+1,t,n}\left(1\right),\ldots ,x_{k+1,t,n}\left(P\Gamma \right)\right]}^{T}$; note that $x_{k+1,t,n}\left(i\right),i\leq \Gamma$ represented the original symbols while $x_{k+1,t,n}\left(i\right),i>\Gamma$ were replicas of the original symbols, which would be forwarded by the relay nodes.

(9) $${\left[v_{1},{\ldots ,v}_{\Gamma }\right]}^{T}={\bar{x}}_{k+1}{X_{k}}^{H}$$

The selected coefficient vector could be represented using (9), such that given all the possible outcomes of ${\bar{x}}_{k+1}$, there existed $2^{\Gamma m}$ corresponding coefficient vectors. In other words, there was a one-to-one mapping between the coefficient vectors and the input bits. Note that there was no difference between the differentially encoded symbols generated by our co-efficient vector design in (8) and those generated by the unitary matrices design in [29,30] for example.

Provided that the differential scheme was designed such that all nodes in the network have prior knowledge of the co-efficient vector set v and the mapping scheme $P\left(\cdot \right)$, the relay nodes could simply recover the elements of the co-efficient vector set using (10) and then using (8) for differential encoding:

(10) $${\left[{\tilde{v}}_{1},{\ldots ,\tilde{v}}_{\Gamma }\right]=\arg min\left|r_{k+1,p}{r_{k,p}}^{T}-{\left|f_{k,p}\right|}^{2}\left[v_{1},{\ldots ,v}_{N}\right]\right|}^{2}$$

where $r_{k+1,p}=\sqrt{E_{S}P\Gamma }x_{k+1}⊙f_{k+1,p}+n_{k+1,p}$ was the signal received at the *p*th relay node in the ${\left(k+1\right)}_{th}$ block. The differential decoding protocol employed by our scheme relied on the assumption that the channel co-efficients remain unchanged for transmissions in consecutive blocks; in other words, $f_{k,p}≅f_{k+1,p}$. In our proposed scheme, the relay nodes only recovered the elements of the co-efficient vector set as implemented in (10), without recovering the original information bits. This approach ensured that the computational complexity of the encoder was reduced because the relay nodes avoided the mapper block and co-efficient vector generator block in Figure 2. To be precise, the number of comparisons c at each relay node was reduced by $1\leq c\leq 2^{Pm}$. This issue is further explained in the simulation section.

We continue our discussion on differential encoding at the relay nodes by illustrating how the co-efficient vector design was implemented with M-PSK constellations for P relays. Note that we assumed that the relay nodes could perfectly compute the elements of the coefficient vector set. Considering an M-PSK modulation scheme with spectral efficiency m, the encoder at each relay node generated Γ modulated signals from Γm information bits. Let the set of recovered information bits at the encoder be given as $b_{l},l=1,2,\ldots ,2^{2m}$, and the co-efficient vector set is computed as: $v\left\{{\left[v_{1,1},{\ldots ,v}_{\Gamma ,1}\right]}^{T},{\left[v_{1,2},{\ldots ,v}_{\Gamma ,2}\right]}^{T},\ldots ,{\left[v_{1,2^{2m}},\ldots ,v_{\Gamma ,2^{2m}}\right]}^{T}\right\}$. Thus, the mapping scheme $P\left(.\right)$ for each set of information bits to a co-efficient vector set was defined by:

(11) $$P\left\{\left(b_{1}\right),\left(b_{2}\right),\ldots ,\left(b_{2^{2m}}\right)\right\}=\left\{{\left[v_{1,1},{\ldots ,v}_{\Gamma ,1}\right]}^{T},{\left[v_{1,2},{\ldots ,v}_{\Gamma ,2}\right]}^{T},\ldots ,{\left[v_{1,2^{2m}},\ldots ,v_{\Gamma ,2^{2m}}\right]}^{T}\right\}$$

### 3.4. Differential Decoding Using Co-Efficient Vectors

Similar to (7), the received signal matrix in the (*k* + 1)th block was given by:

(12) $$Y_{k+1}=\sqrt{\rho }X_{k+1}G_{k+1}+Z_{k+1}$$

where $\rho =\frac{E_{C}E_{S}N_{C}}{E_{S}+1}$, $Y_{k+1}=\left[y_{k,1},\ldots ,y_{k,\Gamma }\right]\in C^{\Gamma \times \Gamma }$, $y_{k,i}={\left[y_{k,i}\left(1\right),\ldots ,y_{k,i}\left(\Gamma \right)\right]}^{T}$, $X_{k+1}=\left[M_{1}{\tilde{\bar{x}}}_{k+1},\ldots ,M_{J}{\tilde{\bar{x}}}_{k+1},M_{J+1}{{\tilde{\bar{x}}}_{k+1}}^{*},\ldots ,M_{P}{{\tilde{\bar{x}}}_{k+1}}^{*}\right]\in C^{\Gamma \times P}$, $G_{k+1}=\left[g_{k+1,1},\ldots ,g_{k+1,\Gamma }\right]\in C^{P\times \Gamma }$, $g_{k+1,i}={\left[g_{k+1,i,t,n}\left(1\right),\ldots ,g_{k+1,i,t,n}\left(P\right)\right]}^{T}=\left(I_{P}⊗\omega^{T}\right)g$, $g={\left[g_{1,t}\left(0\right),\ldots ,g_{1,t}\left(L-1\right),\ldots ,g_{P,t}\left(0\right),\ldots ,g_{P,t}\left(L-1\right)\right]}^{T}$, and $Z_{k+1}=\left[{\tilde{z}}_{k+1,1},\ldots ,{\tilde{z}}_{k+1,\Gamma }\right]\in C^{\Gamma \times \Gamma },z_{k+1,i}={\left[z_{k+1,i}\left(1\right),\ldots ,z_{k+1,i}\left(\Gamma \right)\right]}^{T}$. As far as the destination was concerned, consecutive Γ×P quasi-orthogonal signal matrices $X_{k}$ and $X_{k+1}$ had been received in two consecutive transmission blocks k and k+1 based on (7) and (12). The received signals at the destination could thus be rewritten as:

(13) $$y_{k,i}=X_{k}g_{k,i}+z_{k,i}={\left[\left(M_{p}{\tilde{\bar{x}}}_{k}\right)G_{k}+z_{k,i}\right]}^{T}\;y_{k+1,i}=X_{k+1}g_{k+1,i}+z_{k+1,i}={\left[\left(M_{p}{\tilde{\bar{x}}}_{k+1}\right)G_{k+1}+z_{k+1,i}\right]}^{T}\;i=1,2,\ldots ,\Gamma p=1,..,J,J+1,\ldots ,P$$

Using the signals received in (13) in the *k*th block and (*k* + 1)th block, respectively, the estimate of the elements of the co-efficient vector $\left\{{\tilde{v}}_{1},{\ldots ,\tilde{v}}_{\Gamma }\right\}$ could be recovered pairwisely at the destination without CSI. For example, for a cooperative network with P=4 relay nodes and Γ=4, in order to recover $\left\{{\tilde{v}}_{1},{{\tilde{v}}_{2},\ldots ,\tilde{v}}_{4}\right\}$, we first obtained the quasi-orthogonal signal and channel matrices for two consecutive transmission blocks as follows:

$$X_{q}=\left[\begin{array}{cc} \begin{array}{cc} {\tilde{\bar{x}}}_{q,1,0,0}\left(1\right) & {\tilde{\bar{x}}}_{q,2,0,0}\left(2\right)\\ {-{\tilde{\bar{x}}}_{q,1,0,1}\left(2\right)}^{*} & {{\tilde{\bar{x}}}_{q,2,0,1}\left(1\right)}^{*} \end{array} & \begin{array}{cc} {\tilde{\bar{x}}}_{q,3,0,0}\left(3\right) & {\tilde{\bar{x}}}_{q,4,0,0}\left(4\right)\\ {{-\tilde{\bar{x}}}_{q,3,0,1}\left(4\right)}^{*} & {{\tilde{\bar{x}}}_{q,4,t,0,1}\left(3\right)}^{*} \end{array}\\ \begin{array}{cc} {{-\tilde{\bar{x}}}_{q,1,1,0}\left(3\right)}^{*} & {{-\tilde{\bar{x}}}_{q,2,1,0}\left(4\right)}^{*}\\ {\tilde{\bar{x}}}_{q,1,1,1}\left(4\right) & -{\tilde{\bar{x}}}_{q,2,1,1}\left(3\right) \end{array} & \begin{array}{cc} {{\tilde{\bar{x}}}_{q,3,1,0}\left(1\right)}^{*} & {{\tilde{\bar{x}}}_{q,4,1,0}\left(2\right)}^{*}\\ -{\tilde{\bar{x}}}_{q,3,1,1}\left(2\right) & {\tilde{\bar{x}}}_{q,4,1,1}\left(1\right) \end{array} \end{array}\right]$$

$$G_{q}=\left[\begin{array}{cc} \begin{array}{cc} g_{q,1,0,0}\left(1\right) & {g_{q,2,0,0}\left(2\right)}^{*}\\ g_{q,1,0,1}\left(2\right) & -{g_{q,2,0,1}\left(1\right)}^{*} \end{array} & \begin{array}{cc} {g_{q,3,0,0}\left(3\right)}^{*} & g_{q,4,0,0}\left(4\right)\\ {g_{q,3,0,1}\left(4\right)}^{*} & -g_{q,4,0,1}\left(3\right) \end{array}\\ \begin{array}{cc} g_{q,1,1,0}\left(3\right) & {g_{q,2,1,0}\left(4\right)}^{*}\\ g_{q,1,1,1}\left(4\right) & -g_{q,2,1,1}\left(3\right) \end{array} & \begin{array}{cc} -{g_{q,3,1,0}\left(1\right)}^{*} & -g_{q,4,1,0}\left(2\right)\\ -{g_{q,3,1,1}\left(2\right)}^{*} & g_{q,4,1,1}\left(1\right) \end{array} \end{array}\right]$$

where $X_{q}\in C^{\Gamma \times P}$ and $G_{q}\in C^{P\times \Gamma }$, $q\in \left\{k,k+1\right\}$ were quasi-orthogonal signal and channel matrices, respectively. The *i*th information signal transmitted by the source node, through the *p*th relay node on the *n*th sub-carrier during the *t*th OFDM time slot, was denoted by ${\tilde{\bar{x}}}_{q,p,t,n}\left(i\right)$, and $g_{q,i,t,n}\left(p\right)$ captured the channel gain on the *n*th sub-carrier during *t*th OFDM time slot between the pth relay node and the destination. Since it was assumed that the channel was constant during the transmission of Γ symbols, $g_{q,i,t,n}\left(p\right)$ was therefore constant for i=1,2,…,Γ; similarly, ${\tilde{\bar{x}}}_{q,p,t,n}\left(i\right)$ was constant for p=1,2,…,P since all the cooperating relay nodes transmitted identical information signals. Thus, it could be implied that $g_{q,1,t,n}\left(p\right)=,\ldots ,=g_{q,4,t,n}\left(p\right)=g_{q,t,n}\left(p\right)$ and ${\tilde{\bar{x}}}_{q,1,t,n}\left(i\right)=,\ldots ,={\tilde{\bar{x}}}_{q,4,t,n}\left(i\right)={\tilde{\bar{x}}}_{q,t,n}\left(i\right)$. Based on this, we computed:

$$X_{j}X_{j}^{G}=\left[\begin{array}{cccc} X_{1} & 0 & 0 & X_{2}\\ 0 & X_{1} & -X_{2} & 0\\ 0 & -X_{2} & X_{1} & 0\\ X_{2} & 0 & 0 & X_{1} \end{array}\right]{\;G}_{j}G_{j}^{G}=\left[\begin{array}{cccc} G_{1} & 0 & 0 & G_{2}\\ 0 & G_{1} & -G_{2} & 0\\ 0 & -G_{2} & G_{1} & 0\\ G_{2} & 0 & 0 & G_{1} \end{array}\right]$$

where $X_{1}=\sum_{i=1}^{4}{\left|{\tilde{\bar{x}}}_{q,t,n}\left(i\right)\right|}^{2}$ was the signal power, and $X_{2}=2Re\left({\tilde{\bar{x}}}_{q,t,n}\left(1\right){{\tilde{\bar{x}}}_{q,t,n}\left(4\right)}^{*}-{\tilde{\bar{x}}}_{q,t,n}\left(2\right){{\tilde{\bar{x}}}_{q,t,n}\left(3\right)}^{*}\right)$ was a self-interference parameter. Similarly, $G_{1}=\sum_{p=1}^{4}{\left|g_{q,t,n}\left(p\right)\right|}^{2}$ was the channel power, and $G_{2}=2Re\left\{g_{q,t,n}\left(1\right){g_{q,t,n}\left(4\right)}^{*}-g_{q,t,n}\left(2\right){g_{q,t,n}\left(3\right)}^{*}\right\}$ was a self-interference parameter. The elements of the co-efficient vector ${\bar{v}}_{k+1}=\left[v_{1},{\ldots ,v}_{4}\right]$ were then recovered as follows:

(14) $$\begin{array}{c} y_{k+1,1}{y_{k,1}}^{H}=\left({\tilde{\bar{x}}}_{k+1}{X^{k}}^{H}\right)G_{k+1}{g_{k,1}}^{H}+Z_{1}\\ =\left({\bar{v}}_{k+1}X_{k}{X_{k}}^{H}\right)G_{k+1}{g_{k,1}}^{H}+Z_{1}\\ =v_{1}\left(X_{1}G_{1}+X_{2}G_{2}\right)+v_{4}\left(X_{1}G_{2}+X_{2}G_{1}\right)+Z_{1}\\ =v_{1}A+v_{4}B+Z_{1} \end{array}$$

Similarly,

(15) $$\begin{array}{c} y_{k+1,1}{y_{k,2}}^{H}=\left({\tilde{\bar{x}}}_{k+1}{X^{k}}^{H}\right)G_{k+1}{g_{k,2}}^{H}+Z_{2}\\ =\left({\bar{v}}_{k+1}X_{k}{X_{k}}^{H}\right)G_{k+1}{g_{k,2}}^{H}+Z_{2}\\ =v_{2}A-v_{3}B+Z_{2} \end{array}$$

(16) $$\begin{array}{c} y_{k+1,1}{y_{k,3}}^{H}=\left({\tilde{\bar{x}}}_{k+1}{X^{k}}^{H}\right)G_{k+1}{g_{k,3}}^{H}+Z_{3}\\ =\left({\bar{v}}_{k+1}X_{k}{X_{k}}^{H}\right)G_{k+1}{g_{k,3}}^{H}+Z_{3}\\ =-v_{2}B+v_{3}A+Z_{3} \end{array}$$

(17) $$\begin{array}{c} y_{k+1,1}{y_{k,4}}^{H}=\left({\tilde{\bar{x}}}_{k+1}{X^{k}}^{H}\right)G_{k+1}{g_{k,4}}^{H}+Z_{4}\\ =\left({\bar{v}}_{k+1}X_{k}{X_{k}}^{H}\right)G_{k+1}{g_{k,4}}^{H}+Z_{4}\\ =v_{1}B+v_{4}A+Z_{4} \end{array}$$

where $Z_{n}$ was the noise, $A=X_{1}G_{1}+X_{2}G_{2}$, and $B=X_{1}G_{2}+X_{2}G_{1}$, and we referred to A and B as the differential decoding parameters required to recover ${\bar{v}}_{k+1}$. The differential decoding parameters were computed at the destination as:

(18) $$\begin{array}{c} y_{k,1}{y_{k,4}}^{H}=X_{k}{X_{k}}^{H}g_{k,1}{g_{k,4}}^{H}+{\tilde{Z}}_{4}=A+{\tilde{Z}}_{4}\\ y_{k,1}{y_{k,1}}^{H}=X_{k}{X_{k}}^{H}g_{k,1}{g_{k,1}}^{H}+{\tilde{Z}}_{1}=B+{\tilde{Z}}_{1} \end{array}$$

This implied that $y_{k,1}{y_{k,4}}^{H}\approx A$ and $y_{k,1}{y_{k,1}}^{H}\approx B$ since $Z_{n}\approx {\tilde{Z}}_{n}$. It was thus obvious from (18) that the scheme did not require CSI to recover ${\bar{v}}_{k+1}$. The non-coherent recovery of ${\bar{v}}_{k+1}$ rather depended on consecutively received signals in the *k*th block and (*k* + 1)th block. Once A and B were computed at the destination using (18), the information signals in (14) to (17) can be recovered pair-wisely. Obviously, all the decision signals were only a function of a pair of input signals, which existed with dissimilar constellation angles. This offered the possibility of decoding in pairs. We could decide for each pair of recovered symbols independently using a pair-wise least square decoder as follows:

(19) $$\left({\tilde{v}}_{1},{\tilde{v}}_{4}\right)=\arg\min\left[{\left|y_{k+1,1}{y_{k,1}}^{H}-\left(v_{1}A+v_{4}B\right)\right|}^{2}+{\left|y_{k+1,1}{y_{k,4}}^{H}-\left(v_{1}B+v_{4}A\right)\right|}^{2}\right]$$

(20) $$\left({\tilde{v}}_{2},{\tilde{v}}_{3}\right)=\arg\min\left[{\left|y_{k+1,1}{y_{k,2}}^{H}-\left(v_{2}A-v_{3}B\right)\right|}^{2}+{\left|y_{k+1,1}{y_{k,3}}^{H}-\left(v_{3}A-v_{2}B\right)\right|}^{2}\right]$$

The pair-wise least square decoder performed an exhaustive search over all possible combination of constellation points to determine the pair of signals that minimized the terms in (19) and (20). This decoding was equivalent to finding the minimum Euclidean distance between the noisy received signals and the known constellation points.

The resultant elements of the co-efficient vector in (19) and (20) could be interpreted as noisy versions of elements of the scaled co-efficient vector set. The scaling, A and B, however, have a negligible effect on the geometry of the detection region. Since all the elements of the co-efficient vector set v had equal lengths, the destination selected the closest co-efficient vector to ${\left[{\tilde{v}}_{1},{\ldots ,\tilde{v}}_{\Gamma }\right]}^{T}$ from v as the detector output. Then, inverse mapping was applied to recover the information bits. The complexity of this process was equivalent to $2^{M+1}$ since this was the number of constellation points to be examined.

The pairwise detection algorithm described so far for P=4 could be used for any cooperative network with u relays (2≤u≤P). The only difference in design was in the structure of the channel matrix $G_{k}$ and $G_{k+1}$. In this case, the path gain $g_{q,t,n}\left(p\right),p>u$ between the *p*th relay node and the destination was set to zero. In other words, the design for a 3-relay network could be achieved from the design for a 4-relay network simply by setting the path gain $g_{q,t,n}\left(4\right)$ between the 4th relay and the destination to zero. Similarly, cooperative networks with five, six, and seven relays could derive their designs from the design for 8-relay networks.

## 4. Pairwise Error Probability Analysis

We then proceeded to develop sufficient conditions based on the PEP analysis for our code to achieve full diversity of order PΓ while the coding gain was maximized as much as possible. Since each of the K blocks contained arbitrary symbols, which were independently distributed across the relay nodes, only a single block *k* is required for our PEP analysis, which was valid for any k=1,2,…,K. It was assumed that the path gains $f_{p,t}(l)$ and $g_{p,t}(l)$ were independent for different transmission paths such that the P relay nodes form spatially uncorrelated channels between the source node and the destination. Thus, PΓ information symbols are simultaneously distributed in the spatial, temporal, and frequency dimensions. Based on this, and the analysis of [31], the achievable diversity order of our code could be determined as the product of the number of relay nodes, the rank of the temporal correlation matrix, and the number of delay paths, when sufficient conditions were reached. These conditions were discussed below.

The frequency response vector between the source node and the relay nodes was denoted by $f_{k}={\left[f_{k}\left(1\right),\ldots ,f_{k}\left(P\Gamma \right)\right]}^{T}$. Similarly, the frequency response vector between the relay nodes and the destination was $g_{k}={\left[g_{k,1}(1),\ldots ,g_{k,1}(\Gamma ),\ldots ,g_{k,P}(1),\ldots ,g_{k,P}(\Gamma )\right]}^{T}$. The correlation matrix of the channel frequency response could be found as $R=E\left\{h_{k}{h_{k}}^{H}\right\}=E\left\{\left(f_{k}⊙g_{k}\right){\left(f_{k}⊙g_{k}\right)}^{H}\right\}$. Unlike the case of multiple antenna systems, the cooperative network had the ‘transmit’ and ‘cooperate’ stages; thus, R could be decomposed as $R=R_{1}⊙R_{2}$. It can easily be shown that R, $R_{1}$**,** and $R_{2}$ were full rank based on the following:

(21) $$\begin{array}{cc} R_{1}=E\left\{f_{k}{f_{k}}^{H}\right\} & =W_{1}E\left\{f_{p,t}{f_{p,t}}^{H}\right\}{W_{1}}^{H}\\ & =W_{1}diag\left({\sigma_{SR}}^{2}\left(0\right),\ldots ,{\sigma_{SR}}^{2}(L_{SR}-1)\right){W_{1}}^{H} \end{array}$$

(22) $$\begin{array}{cc} R_{2}=E\left\{g_{k}{g_{k}}^{H}\right\} & =W_{2}E\left\{g_{p,t}{g_{p,t}}^{H}\right\}{W_{2}}^{H}\\ & =W_{2}diag\left({\sigma_{RD}}^{2}\left(0\right),\ldots ,{\sigma_{RD}}^{2}(L_{RD}-1)\right){W_{2}}^{H} \end{array}$$

(23) $$W_{1}=\left[{w^{\alpha_{0}}}^{T},\ldots ,{w^{\alpha_{L-1}}}^{T}\right],W_{2}=\left[{w^{\beta_{0}}}^{T},\ldots ,{w^{\beta_{L-1}}}^{T}\right],\;w=\left[1,\omega^{1},\ldots ,\omega^{\left(P\Gamma -1\right)}\right],{\omega =e}^{-j2\pi \Delta f}$$

where $f_{p,t}={\left[f_{p,t}\left(0\right),\ldots ,f_{p,t}\left(L_{SR}-1\right)\right]}^{T}$ and $g_{p,t}={\left[g_{p,t}\left(0\right),\ldots ,g_{p,t}\left(L_{RD}-1\right)\right]}^{T}$, and Δf=1/T was the sub-carrier spacing. From (23), if $W_{1}$ and $W_{2}$ were unitary matrices (valid if all $L_{SR}$ and $L_{RD}$ fall within the sampling instances of the relay nodes and destination respectively [31], then $W_{1}$ and $W_{2}$ have full rank. Furthermore, based on the theorem in Section 1.2.4 of [32], which stated that; if $R_{1}$ and $R_{2}$ were positive definite, then R was itself a positive definite (full rank correlation matrix), R, $R_{1}$**,** and $R_{2}$ could thus be verified as positive definite (full rank correlation matrices).

Since it was established that R had full rank, we then proceeded to discuss the criteria in order to achieve maximum diversity. Statistically independent samples of the source-relay channel were defined as $f=\left[f_{1,t}\left(0\right),\ldots ,f_{1,t}\left(L_{SR}-1\right),\ldots ,f_{P,t}\left(0\right),\ldots ,f_{P,t}\left(L_{SR}-1\right)\right]$. Similarly, statistically independent samples of the relay–destination channel were defined as $g=\left[g_{1,t}\left(0\right),\ldots ,g_{1,t}\left(L_{RD}-1\right),\ldots ,g_{P,t}\left(0\right),\ldots ,g_{P,t}\left(L_{RD}-1\right)\right]$. Under the assumption that all $f_{p,t}\left(l\right)$ and $g_{p,t}\left(l\right)$ were independent identically distributed complex Gaussian variables, it could be implied that $h=\left[h_{1,t}\left(0\right),\ldots ,h_{1,t}\left(L-1\right),\ldots ,h_{P,t}(0),\ldots ,h_{P,t}(L-1)\right],h_{p,t}(l)=f_{p,t}\left(l\right)\cdot g_{p,t}\left(l\right)$. For any $k_{th}$ block, the STF codeword could be viewed as a collection of symbols transmitted across Γ time/frequency slots by P relay nodes. Based on this, the consecutively received signals at the destination in the $k_{th}$ block and ${\left(k+1\right)}_{th}$ block could be rewritten as (24) under the constraint that the sub-channel gain of adjacent transmission blocks was almost constant.

(24) $$\begin{array}{c} Y^{k}={\bar{X}}^{k}\Lambda h+Z^{k}\\ Y^{k+1}={\bar{X}}^{k+1}\Lambda h+Z^{k+1} \end{array}$$

where $Y^{k}={\left[y_{1}^{k},\ldots ,y_{\Gamma }^{k}\right]}^{T}$, $y_{i}^{k}={\left[y^{k}(1),\ldots ,y^{k}(\Gamma )\right]}^{T}$, $Y^{k+1}={\left[y_{1}^{k+1},\ldots ,y_{\Gamma }^{k+1}\right]}^{T}$, $y_{i}^{k+1}={\left[y^{k+1}(1),\ldots ,y^{k+1}(\Gamma )\right]}^{T},\;{\bar{X}}^{k}=diag\left[x_{k,1},\ldots ,x_{k,\Gamma }\right]$, $x_{k,i}=\left[x_{k,n}(1),\ldots ,x_{k,n}(P)\right]$, ${\bar{X}}^{k+1}=diag\left[x_{k+1,1},\ldots ,x_{k+1,\Gamma }\right]$, $x_{k+1,i}=\left[x_{k+1,i}(1),\ldots ,x_{k+1,i}(P)\right],\Lambda ={\left[\Lambda (1),\ldots ,\Lambda (\Gamma )\right]}^{T}$, $\Lambda \left(i\right)=I_{P}⊗\omega^{T}$, $\omega ={\left[1,e^{-j2\pi n/N},\ldots ,e^{-j2\pi L-1n/N}\right]}^{T}$. Using the following notations:

$Y={\left[{Y^{k}}^{T},{Y^{k+1}}^{T}\right]}^{T},V^{k+1}=diag\left[v_{k+1,1},\ldots ,v_{k+1,\Gamma }\right],v_{k+1,i}=\left[v_{k+1,i}(1),\ldots ,v_{k+1,i}(P)\right],X=\left[{I_{P\Gamma }}^{T},{V^{k+1}}^{T}\right],Z={\left[{Z^{k}}^{T},{Z^{k+1}}^{T}\right]}^{T},$ and the recursion ${\bar{X}}^{k+1}=\left\{\begin{array}{c} V^{k+1}{\bar{X}}^{k},k\geq 1\\ I_{P\Gamma },k=0 \end{array}\right..$

We could write:

(25) Y=XWh+Z

The conditional probability density function of the receive signal matrix Y was:

(26) $$p\left(Y|V^{k+1}\right)=\frac{exp\left(-tr\left\{Y{\left(I_{P\Gamma }+\Upsilon X\Lambda R{\Lambda^{H}X}^{H}\right)}^{-1}Y^{H}\right\}\right)}{\pi^{P\Gamma }det\left(I_{P\Gamma }+\Upsilon X\Lambda R{\Lambda^{H}X}^{H}\right)}$$

where $C_{v}=\left(I_{P\Gamma }+\Upsilon X\Lambda R{\Lambda^{H}X}^{H}\right)$ was the covariance matrix of Y, tr denoted the trace function, and Υ was the average SNR of the received signal given as $\Upsilon =\frac{P\Upsilon_{SR}\Upsilon_{RD}}{1+\Upsilon_{SR}+P\Upsilon_{RD}}$. Thus, the non-coherent ML decoder was given by:

(27) $${\hat{V}}^{k+1}=\arg\underset{V^{k+1}\in V}{\max}p\left(Y|V^{k+1}\right)$$

Substituting $Y^{k}$ into $Y^{k+1}$ in (24) and using ${\bar{X}}^{k+1}=V^{k+1}{\bar{X}}^{k}$, we had $Y^{k+1}=V^{k+1}Y^{k}+{\overset{`}{Z}}^{k+1}$, where ${\overset{`}{Z}}^{k+1}=Z^{k+1}-V^{k+1}Z^{k}$. The non-coherent ML decoder could thus be simplified as:

(28) $${\hat{V}}^{k+1}=\arg\underset{V^{k+1}\in V}{\max}\left\|Y^{k}+{V^{k+1}}^{H}Y^{k+1}\right\|$$

where $\left\|\cdot \right\|$ was the Frobenius norm. The Chernoff bound on the PEP of mistaking $V^{k+1}$ by ${\overset{´}{V}}^{k+1}$ could be given as [33].

(29) $$PEP\left(V^{k+1}-{\overset{´}{V}}^{k+1}\right)=\frac{1}{2}\left\{\frac{\det\left[\lambda \left(I_{P\Gamma }+\Upsilon X\Lambda R{\Lambda^{H}X}^{H}\right)+\left(1-\lambda \right)\left(I_{P\Gamma }+\Upsilon \overset{´}{X}\Lambda R\Lambda^{H}{\overset{´}{X}}^{H}\right)\right]}{\overset{\lambda }{\det}\left(I_{P\Gamma }+\Upsilon X\Lambda R{\Lambda^{H}X}^{H}\right){\cdot \det}^{\left(1-\lambda \right)}\left(I_{P\Gamma }+\Upsilon \overset{´}{X}\Lambda R\Lambda^{H}{\overset{´}{X}}^{H}\right)}\right\}$$

where X and $\overset{´}{X}$ were two distinct codewords, $\overset{´}{X}=\left[{I_{P\Gamma }}^{T},{{\overset{´}{V}}^{k+1}}^{T}\right]$ and $\lambda =E\left\{\exp\left(\lambda \left[lnp\left(Y|V^{k+1}\right)-p\left(Y|{\overset{´}{V}}^{k+1}\right)\right]\right)\right\}$ was used to get the tightest bound. By simple algebraic manipulation (29) could be simplified as

(30) PEP(V^(k+1)−V´^(k+1))=1/2{det⁡[I_PΓ+ΥΛRΛ^H(XλX^H+X´(1−λ)X´^H)]/det⁡[I_PΓ+2ΥΛRΛ^H]}

Since the relay nodes in our scheme linearly processed their received signals, our achievable diversity order was bounded by $L=min\left\{L_{SR},L_{RD}\right\}$ and $\tau =min(\tau_{SR},\tau_{RD})$ where τ was the rank of the channel temporal correlation matrix. Thus, targeting maximum diversity order Γ=Lτ was chosen. Other values of Γ may be desirable, for example, when targeting minimum decoding complexity or when high SNR is considered. It could be deduced from (30) that for all values of k, if ${\overset{´}{V}}^{k+1}-V^{k+1}$ or similarly if $\overset{´}{X}-X$ had full rank, then our scheme would achieve a diversity order of PLτ. At high SNR, the term in (30) could be further bounded as (31), where λ=1/2 was selected to get the tightest bound [27].

(31) $$PEP\left(V^{k+1}-{\overset{´}{V}}^{k+1}\right)\leq {\left(\frac{\Upsilon }{8}{\left(\det\left(\Lambda R\Lambda^{H}\right)\det\left[{\left({\overset{´}{V}}^{k+1}-V^{k+1}\right)}^{H}\left({\overset{´}{V}}^{k+1}-V^{k+1}\right)\right]\right)}^{\frac{1}{PL}}\right)}^{-PL}$$

From (31), it was observed that the performance of our code was determined by R,$\Lambda^{H}\Lambda$ and ${\left({\overset{´}{V}}^{k+1}-V^{k+1}\right)}^{H}\left({\overset{´}{V}}^{k+1}-V^{k+1}\right)$. It has already been established that R had full rank; thus, our scheme would achieve maximum diversity strictly on the condition that $\Lambda^{H}\Lambda$ and ${\left({\overset{´}{V}}^{k+1}-V^{k+1}\right)}^{H}\left({\overset{´}{V}}^{k+1}-V^{k+1}\right)$ had full rank. Since of our main focus was to maximise diversity whilst ensuring maximum coding gain, the code had to be designed such that ${\overset{´}{V}}^{k+1}-V^{k+1}$ had full rank PΓ over all possible pairwise errors. When maximum diversity was achieved, ${\overset{´}{V}}^{k+1}-V^{k+1}$ had full rank; the coding gain was only determined by $\det\left(\Lambda^{H}\Lambda \right)$ and $\det\left[{\left({\overset{´}{V}}^{k+1}-V^{k+1}\right)}^{H}\left({\overset{´}{V}}^{k+1}-V^{k+1}\right)\right]$. In order to maximize the coding gain, the first step was to provide PΓ uncorrelated channels such that $\det\left(\Lambda^{H}\Lambda \right)$ was maximized. For the second step, we considered the diversity product $\zeta_{c}$, which measured the quality of the code given as $\zeta_{c}=\frac{1}{2}\underset{{\overset{´}{V}}^{k+1}\neq V^{k+1}\forall V}{\min}{\left|\det\left({\overset{´}{V}}^{k+1}-V^{k+1}\right)\right|}^{\frac{1}{PL}}$ where $\zeta_{c}>0$ achieved maximum diversity. Thus, the coding gain was maximized when we maximized $\zeta_{c}$ under the constraint that: $0\leq \zeta_{c}\leq 1$ and $\left({\overset{´}{V}}^{k+1}-V^{k+1}\right),\forall {\overset{´}{V}}^{k+1}\neq V^{k+1}$.

## 5. Performance Evaluation

**Figure 3:**
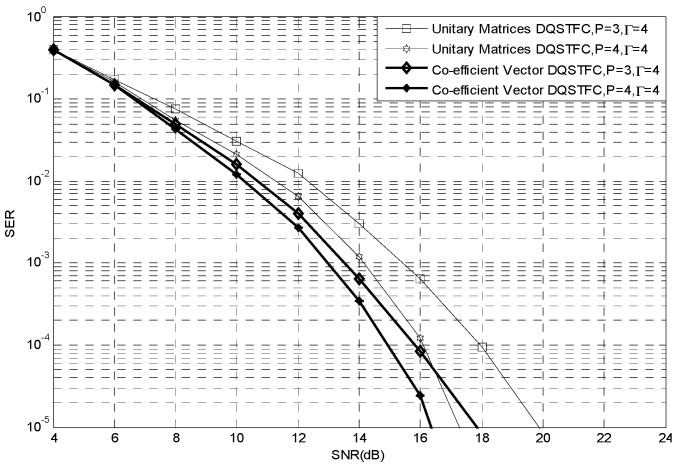
Comparison of Unitary Matrices and Co-efficient Vector Designs.

The performance of the co-efficient vector differential DQSTBC scheme with cooperating relay nodes over severely fading multi-path channels is evaluated via simulation. The fading is assumed to vary identically during the transmission of at least two consecutive information blocks. Here, it is assumed that the cooperating nodes [34,35,36] and the destination are unable to acquire CSI. Thus, they recover the transmitted bits from consecutively received signals. We set P=4 and m=1 to form the basis for comparing the BER performance of our co-efficient vector design with that of the unitary matrices design. So far in this study, our literature search for a differential DQSTFC scheme for comparison has been unsuccessful. We thus independently simulate the differential DQSTFC scheme using the unitary matrices design and the co-efficient vector design. Also compared is the differential orthogonal scheme of [29], where the unitary matrix design is utilized, with the co-efficient vector based differential orthogonal scheme. A similar DQSTFC scheme is also independently simulated for P=4 cooperating nodes in coherent networks that can acquire perfect CSI. The results for BPSK are presented in Figure 3.

From the results of the simulated schemes, it is evident that, compared to the unitary matrices AF protocol, the performance of the differential DF scheme using co-efficient vectors is slightly better than that obtained using unitary matrices in the low SNR region while the performance improves significantly in the high SNR region. For example, at $10^{-4}$ BER, the performance gain is about 1.2 dB.

A notable difference between the designs is observed in terms of the computation complexity of the decoder at the relay nodes and the destination. For designs utilizing unitary matrices, a simple pairwise decoder recovers the information bits at the relays and the destination by conducting ${c=2}^{m}$ comparisons, m is the number of bits. In contrast, designs utilizing co-efficient vectors rely on an exhaustive search over all possible elements of the co-efficient vector. The most complex method is to perform a full search over all possible combinations in the co-efficient vector set. This equates to $c=2^{2m}$ comparisons to recover the elements of the co-efficient vector set, with a further ${1\leq c\leq 2}^{2m}$ comparisons required for mapping and inverse mapping at the encoder and decoder, respectively. It is thus clear that the decoding complexity limitation of the co-efficient vector based design is due to the constellation size. To counter this, suitable optimum detection schemes, such as those proposed in [37,38], which are independent of constellation size, can be utilized.

Next, we analyse the performance of our proposed scheme in frequency-selective and time-selective channels. Specifically, we set our simulation parameters for different Doppler frequencies $f_{D}$ ranging from 40 Hz to 200 Hz and different root mean square delay spreads $\tau_{rms}$ between 0.1 μs and 4 μs, the Doppler frequencies correspond to mobile speeds between 22 km/h and 108 km/h. For the quasi-orthogonal codes, the rotation angles are set to θ/M, where M is the constellation size.

For different channel conditions, the performance of our proposed differential DQSTFC scheme is compared with the differential quasi-orthogonal DSTC-OFDM and DSFC schemes (whose parameters are simulated in our environment). We study the effects of Doppler spread and delay spread on the aforementioned coding schemes using different simulation parameters. We first set $\tau_{rms}$ to a low value of 0.1 μs such that the effect of delay spread is negligible. To study the influence of Doppler spread, we investigate BER performance at different Doppler frequencies. The SNR is fixed at 12 dB, the symbols are chosen from a QPSK constellation, and all the coding schemes have the same transmission rate of 2 bits/s/Hz.

**Figure 4:**
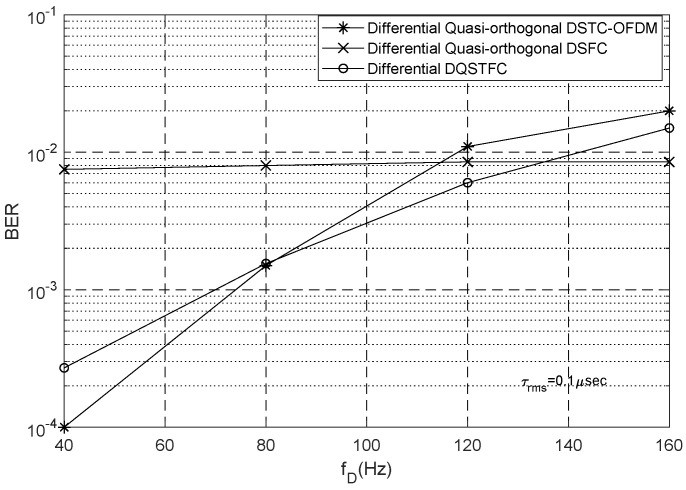
Co-efficient Vector DQSTFC in Frequency Selective Fading Channels.

From Figure 4, it can be seen that for values of $f_{D}$ between 78 Hz and 135 Hz, the BER performance of our proposed differential DQSTFC scheme is better than that of the differential quasi-orthogonal DSTC-OFDM and DSFC schemes. This is because at such values of $f_{D}$ the coherence time $t_{c}$ and coherence bandwidth $b_{c}$ of the channel are large enough to satisfy the requirements of constant channel gain across adjacent time slots and adjacent subcarriers. The BER performance of DSTC-OFDM is better than that of our proposed scheme only at values of $f_{D}$ below 78 Hz. At such values of $f_{D}$, the coherence time is large, and $t_{c}\geq \Gamma$ can be satisfied. Thus, DSTC-OFDM schemes, which require constant channel gain in the temporal dimension for the entire duration of Γ=4 symbols, have the best BER performance. When the Doppler spread increases and $f_{D}$ is between 78 Hz and 135 Hz, the coherence time reduces and $t_{c}\geq \Gamma$ can no longer be satisfied; however, the coherence time is still large enough to satisfy $t_{c}\geq \Gamma /2$. Thus, the BER performance of DSTC-OFDM degrades beyond that of our proposed scheme. When the Doppler spread is severe and $f_{D}$ is above 135 Hz, coding in the temporal dimension introduces a significant amount of inter-symbol interference; thus, our proposed scheme and the DSTC-OFDM scheme experience performance degradation. However, the BER performance of the DSFC scheme is better in this situation because the delay spread is low and the coherence bandwidth is large enough to satisfy $b_{c}\geq \Gamma$. Thus, DSFC schemes that require constant channel gain in the frequency dimension for the entire duration of Γ=4 symbols has the best BER performance. In contrast with DSTC-OFDM and DSFC schemes, where the requirements for constant channel gain must be satisfied for the entire duration of Γ=4 symbols, our scheme only requires constant channel gain during the transmission of Γ=2 symbols. Therefore, we can conclude that for cooperative networks operating in environments where CSI cannot be acquired, our proposed scheme is robust against a practical range of Doppler spread.

**Figure 5:**
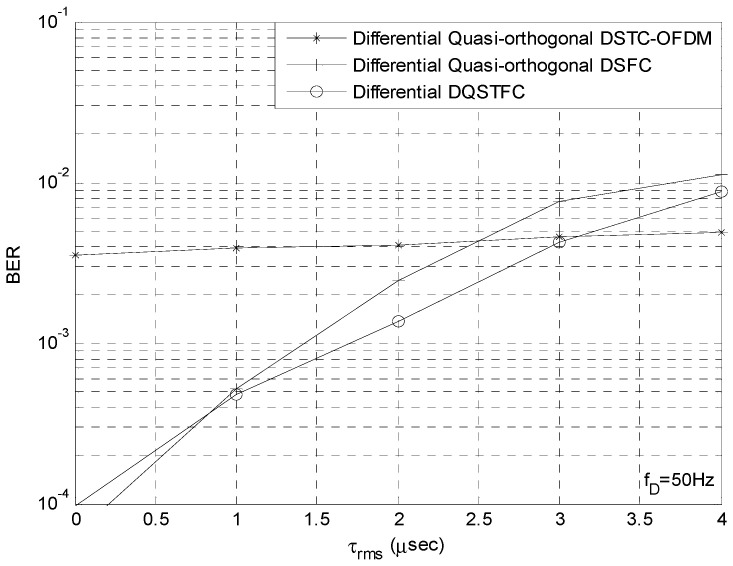
Performance comparison between proposed Co-efficient Vector DQSTFC, DSTC-OFDM and DSFC in Time Selective Fading Channels.

We then set $f_{D}$ to a fixed value of 50 Hz such that the influence of Doppler spread is low and compare the performance of all the aforementioned coding schemes at different levels of delay spread. The SNR is fixed at 12 dB, and all the coding schemes have identical transmission rate of 2 bits/s/Hz. From Figure 5, we observe that our proposed scheme outperforms the other coding schemes when the value of $\tau_{rms}$ is between 0.8 μs and 3.2 μs. This is because at such values, the coherence time and coherence bandwidth of the channel is large enough to satisfy the requirements of constant channel gain across adjacent time slots and adjacent sub-carriers. When the delay spread is low and $\tau_{rms}$ is below 0.8 μs, the coherence bandwidth is large and $b_{c}\geq \Gamma$ can be satisfied. Thus, DSFC schemes that require constant channel gain across Γ sub-carriers outperform the DSTC-OFDM scheme and our proposed DQSTFC scheme. When the delay spread increases, the coherence bandwidth reduces and $b_{c}\geq \Gamma$ can no longer be satisfied; however, $b_{c}\geq \Gamma /2$ can still be satisfied. Thus, our proposed scheme exhibits improved BER performance compared to the DSFC scheme. When $\tau_{rms}$ is higher than 3.2 μs, the coherence bandwidth is very low such that coding in the frequency dimension introduces inter-carrier interference; thus, the BER performance of our proposed scheme and the DSFC scheme degrade significantly. In this condition, however, DSTC-OFDM outperforms the other schemes.

## 6. Conclusions

As opposed to traditional unitary matrix-based differential designs amenable to the AF cooperative protocol, we propose co-efficient vector based differential designs for DF cooperative networks where the relay nodes are required to differentially decode and re-transmit information signals. We employ full rate full diversity quasi-orthogonal codes to meet the demands of high data rate transmission. We present the generalized STF mapping scheme and differential recipe for utilizing co-efficient vectors in cooperative networks with any number of relays. Through PEP analysis, we derive sufficient design criteria for our scheme to achieve full spatial, temporal, and frequency diversity. Using simulation results, we compare and contrast the unitary matrices and co-efficient vector designs in terms of computational complexity and BER performance.

From the results, it is evident that, compared to the unitary matrices AF protocol, the performance of the differential DF scheme using co-efficient vectors is slightly better than that obtained using unitary matrices in the low SNR region while the performance improves significantly in the high SNR region. For example, at $10^{-4}$ BER, the performance gain is about 1.2 dB. Additionally, it can be seen that for values of $f_{D}$ between 78 Hz and 135 Hz the BER performance of our proposed differential DQSTFC scheme is better than that of the differential quasi-orthogonal DSTC-OFDM and DSFC schemes.

We then study the performance of our differential DQSTFC scheme in severe multipath fading conditions. When $\tau_{rms}$ is higher than 3.2 μs, the coherence bandwidth is very low, such that coding in the frequency dimension introduces inter-carrier interference; thus, the BER performance of our proposed scheme and the DSFC scheme degrade significantly. In this condition, however, DSTC-OFDM outperforms the other schemes.

### Future Work

The reconfigurable intelligent surface (RIS) technology has generated considerable interest due to its advantages of low cost, easy deployment, and high controllability [39,40,41]. To further attempt to improve the performance of our proposed co-efficient vector differential DQSTFC scheme under different channel conditions, a RIS-assisted differential DQSTFC scheme will be investigated.

## Data Availability

Data sharing not applicable.
